# Design, Simulation, and Evaluation of Polymer-Based Microfluidic Devices via Computational Fluid Dynamics and Cell Culture “On-Chip”

**DOI:** 10.3390/bios13070754

**Published:** 2023-07-22

**Authors:** Nurzhanna Bakuova, Sultanali Toktarkan, Darkhan Dyussembinov, Dulat Azhibek, Almas Rakhymzhanov, Konstantinos Kostas, Gulsim Kulsharova

**Affiliations:** 1 Department of Electrical and Computer Engineering, School of Engineering and Digital Sciences, Nazarbayev University, Astana 010000, Kazakhstan; nurzhanna.bakuova@nu.edu.kz (N.B.); sultanali.toktarkan@nu.edu.kz (S.T.); dulat.azhibek@nu.edu.kz (D.A.); 2 Department of Mechanical and Aerospace Engineering, School of Engineering and Digital Sciences, Nazarbayev University, Astana 010000, Kazakhstan; darkhan.dyussembinov@nu.edu.kz (D.D.); konstantinos.kostas@nu.edu.kz (K.K.); 3 Nanofabrication Core Lab, King Abdullah University of Science and Technology (KAUST), Thuwal 23955-6900, Saudi Arabia; almas.rakhymzhanov@kaust.edu.sa

**Keywords:** microfluidics, simulation, computational fluid dynamics, liver-on-a-chip, hepatocytes

## Abstract

Organ-on-a-chip (OoC) technology has experienced exponential growth driven by the need for a better understanding of in-organ processes and the development of novel approaches. This paper investigates and compares the flow behavior and filling characteristics of two microfluidic liver-on-a-chip devices using Computational Fluid Dynamics (CFD) analysis and experimental cell culture growth based on the Huh7 cell line. The conducted computational analyses for the two chips showed that the elliptical chamber chip proposed herein offers improved flow and filling characteristics in comparison with the previously presented circular chamber chip. Huh7 hepatoma cells were cultured in the microfluidic devices for 24 h under static fluidic conditions and for 24 h with a flow rate of 3 μL·min^−1^. Biocompatibility, continuous flow, and biomarker studies showed cell attachment in the chips, confirming the cell viability and their consistent cell growth. The study successfully analyzed the fluid flow behavior, filling characteristics, and biocompatibility of liver-on-a-chip prototype devices, providing valuable insights to improve design and performance and advance alternative methods of in vitro testing.

## 1. Introduction

Drug development is a costly and inefficient process with a low overall success rate. It was estimated that the average cost of bringing a new drug to market is approximately USD 1 billion [1,2]. Obtaining human-relevant information at the start of the drug development process is critical for reducing overall time and costs. Although animal models play a crucial role, they cannot provide completely accurate results due to significant differences in drug metabolism pathways among species [3]. Traditional in vitro models based on cultured cells also exhibit different responses to drugs due to the absence of physiological cues present in living tissues [4]. Furthermore, these in vitro models cannot replicate organ–organ interactions since they only consist of a monoculture of cells in a Petri dish [1]. Therefore, alternative in vitro systems are needed to improve the efficiency of drug testing procedures.

Microfluidics-based technologies offer several advantages, including reduced sample volume, detection time, costs, portability, disposability, and integration with sensors for online detection [5]. Among many applications of microfluidics, organ-on-a-chip platforms have a great potential to overcome current challenges in drug testing and become an alternative promising model to traditional in vitro and animal testing [1,6,7]. Organ-on-a-chip platforms comprise multi-channel 3D microfluidic chips used for mimicry of a certain organ utilizing human cell culture. They allow for control of the applied medium flow, hence emulating more closely the corresponding physiological conditions [8]. Additionally, the composition of the circulating medium can be modified, during flow experiments, to achieve an appropriate fluid-to-cell ratio [9], which is not possible in traditional 2D cell culture platforms.

In the last decade, much effort has been directed to developing single-organ models such as liver-on-a-chip (LoC) platforms [10,11,12,13,14,15], as the role of the liver in drug testing is inarguably significant [16]. Apart from biocompatibility of the materials used to fabricate LoC devices and the cell types grown in them, platform design considerations are equally important. Flow conditions in such microfluidic platforms are critical for successful cell growth, culturing, and biomarker analysis. Therefore, cost-effective evaluation of flow conditions within a given design and/or among different alternative designs is crucial for the development of useful microfluidic devices. For example, design layouts that hinder fluid flow to all tissue compartments should be avoided when considering studies of drug/metabolite profiles over time. Additionally, low cell numbers and media volume can negatively affect analytical detection.

Computational Fluid Dynamics (CFD) is a powerful computational method which can be also applied in fluid flow modeling in microchannels. CFD modeling is especially important in microfluidics as complicated layouts with narrow channels can easily result in complex flow patterns that are difficult to predict analytically. CFD can be used to assess and optimize microfluidic devices with respect to both fluid flows and embedded particle motions [17]. Computational simulations can be used for both qualitative and quantitative assessment of flow behavior and air entrapment, which are both important for the case studied herein. Experimentally verified simulation approaches can be subsequently used to cost-effectively forecast nutrient flow and efficiency of the liver-on-a-chip model, ultimately permitting its design optimization.

**Figure 1:**
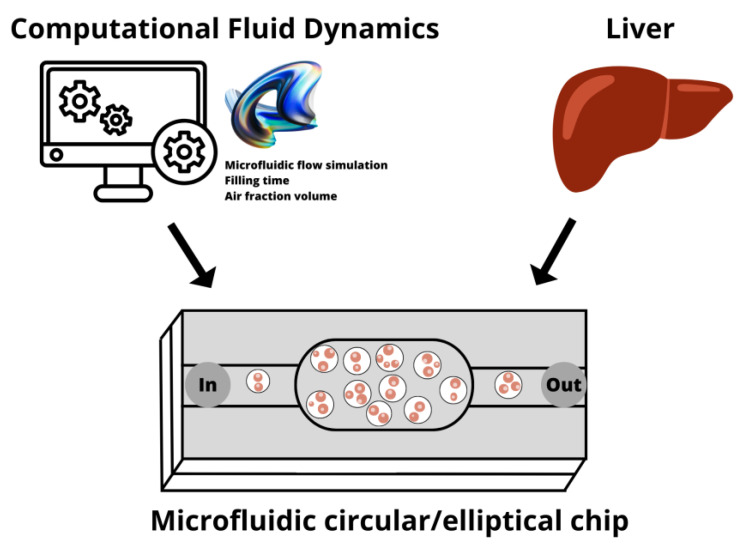
Schematic representation of hepatocyte growth in microfluidic devices modeled and investigated using computational fluid dynamics tools. Image made in canva.com.

We have previously presented in [13] a prototype of a hybrid cyclic-olefin copolymer and poly(dimethyl)sulfoxide-based microfluidic platform for the culture of hepatocytes. The device exhibited excellent biocompatibility and allowed successful hepatocyte culture over time under continuous flow. However, experimental studies revealed that the design of its central cell chamber presented a series of challenges, including air bubble entrapment, flow hindrance, and uneven distribution of cells and media during filling. Therefore, an alternative design with an appropriately adjusted cell chamber is required. This study compares the performance of the previously reported circular microfluidic chip [13] with an alternative design employing an elliptical chamber (Figure 1). Both chip designs are initially assessed using CFD simulations, followed by prototype chip fabrication for experimental verification and evaluation of the viability of seeded cells under static and continuous flow conditions.

Microfluidic device fabrication, flow regime identification, and static and perfusion cell culture analysis along with the relevant techniques employed in this study are discussed first. CFD analysis results for the two designs are presented and evaluated. We also discuss biocompatibility and present the results of experimental cell culture growth in the fabricated microfluidic devices under static conditions. Finally, biomarker studies, conducted under continuous flow in both devices, are compared and discussed.

## 2. Materials and Methods

The materials and methods employed in the design, simulation, fabrication, and experimental verification of the microfluidic devices are briefly presented in this section.

### 2.1. Microfluidic Device Design and Fabrication

**Figure 2:**
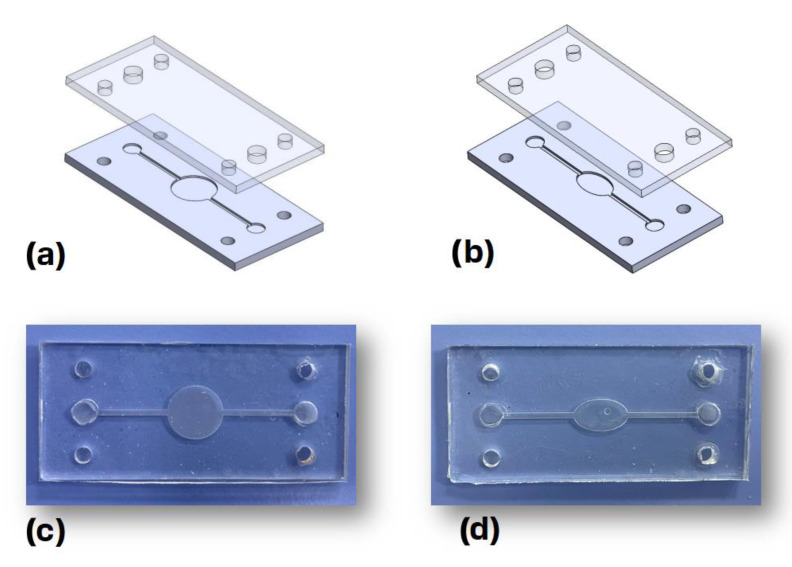
3D models of (**a**) circular and (**b**) elliptical designs and photographs of (**c**) circular and (**d**) elliptical microfluidic devices made of cyclic-olefin copolymer (COC) and polydimethylsiloxane (PDMS).

The Dassault Systemes Solidworks (2021, Student Access 2021–2022) computer-aided design (CAD) software package was used to design the microfluidic devices. The bottom and top parts of the chips, depicted in Figure 2a,b, have principal dimensions of 2.5 cm × 5.5 cm × 0.2 cm. The bottom part of the chips is made out of cyclic-olefin copolymer (COC) featuring two identical microchannels, with a width of 1 mm and a depth 0.5 mm. These two microchannels meet at the central chamber, which is circular for the first design and elliptical for the second one. These bottom parts were fabricated using a milling machine out of COC microscopy slides (ChipShop, Jena, Germany). 

The top part of each chip was fabricated with polydimethylsiloxane (PDMS) via casting. The PDMS base and curing agent were mixed at a ratio of 10:1 and cured at 60 °C for 24 h, while the inlet and outlet holes were then cut using a special puncher. The thermoplastic bonding method presented in [18] was employed to bond the top and bottom parts of the chip. Briefly, the bottom part was treated with corona discharge for 1.5 min before being immersed in a 5% solution of 3-aminopropyl triethoxysilane (APTES) for 20 min, whereas the top part was treated with corona discharge for 1.5 min, rinsed with deionized water, dried, and then bonded to the bottom part through pressure application. The final fabricated chips are depicted in Figure 2c,d, respectively.

### 2.2. Flow Regime Identification

The Reynolds number, a dimensionless number corresponding to the ratio between inertial and viscous forces [19], was calculated to identify the type of flow in the studied microfluidic devices. Flows with Reynolds numbers below 2000 and those that are above 4000 are typically considered laminar and turbulent, respectively [19]. The transient region occurs between these two threshold values. The Reynolds number is defined as:

(1) $$Re=\frac{\rho uL}{\mu }$$

where 𝐿 is a characteristic linear dimension, *u* is the flow speed, ρ is the fluid density, and *μ* the dynamic viscosity. In the case of flows in non-circular tubes and channels, the hydraulic diameter is used as the characteristic length [20]. The hydraulic diameter (*D_H_*) is defined as:

(2) $$D_{H}=\frac{4A}{P}$$

where *A* is the cross-sectional area, and *P* is the wetted perimeter of the cross-section. For the case of water in organ temperature with a flow rate of 15 μL·min^−1^, the Reynolds number is approximately equal to 0.36, which clearly belongs to the laminar regime. This value can be applied to both chip designs since the dimensions of the cross-sections of the microchannels in both designs are identical.

### 2.3. Computational Domain and Boundary Conditions

**Figure 3:**
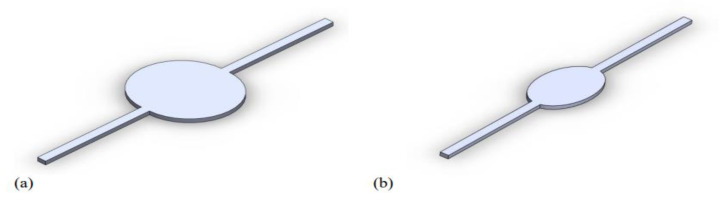
3D CAD models of the computational domain for (**a**) circular and (**b**) elliptical microfluidic devices.

The circular and elliptical chambers of the liver-on-a-chip devices were designed with the SolidWorks software package, as previously mentioned. The regions of interest, corresponding to the computational domains, are depicted in Figure 3. Both designs feature channels with a depth of 0.50 mm and a width of 1 mm, but with a total length of 40 mm and 36 mm for the elliptical and circular designs, respectively. The ellipsis in the elliptical design has a major axis of 10 mm and a minor axis of 6 mm, resulting in a total domain volume of 38.61 cubic milliliters (3.861 × 10^−5^ L^3^). The circular chamber has a diameter of 10 mm, with its total volume reaching 57.27 cubic milliliters (5.727 × 10^−5^ L^3^).

Computational simulations were performed using both COMSOL Multiphysics and ANSYS Fluent for comparison and verification purposes. In COMSOL, the ‘Laminar flow (spf)’ and ‘Transport of Diluted Species (tds)’ interfaces were employed. The model is initially filled with air, water from the inlet at the edge of the microchannel enters with a flow rate of 5/10/15 µL/min (0.83 × 10^−10^/1.67 × 10^−10^/2.5 × 10^−10^ m^3^/s), and 0 gauge pressure is set at the outlet. In ANSYS, the Volume of Fluid multiphase model was employed along with a laminar model in simulations. Through exploitation of the longitudinal symmetry, one half of the computational domain was considered to decrease the required computational time. As described above, the computational domain is initially set to be filled with air only, with water entering, at the start of the simulation, from the device inlet. The equivalent boundary conditions are set in this case. For example, the 15 µL/min flow rate corresponds to an inlet water velocity of 0.0005 m/s. Therefore, the resulting velocity is set at the inlet, a 0 Pa gauge pressure is set at the outlet, and no-slip boundary conditions are applied on the remaining channel walls.

### 2.4. Experimental Setup

#### 2.4.1. Cell Culture

The Huh7 hepatoma cell line (ATCC) was cultured in Dulbecco’s modified Eagle’s medium (DMEM) supplemented with 10% fetal bovine serum (FBS) and 1% penicillin-streptomycin. The cells were maintained in a humidified incubator at 37 °C with 5% CO_2_. After 24 h, cells were used for the flow experiments. The control batches were cultured on collagen-coated 24-well plates using the same density of cell seeding as in the flow experiments.

#### 2.4.2. Cell Seeding and Growth

The cells were seeded into the microfluidic devices under aseptic conditions. Channels were preliminarily washed with 70% ethanol and 1× phosphate-buffered saline (PBS). A 0.01% collagen Type I solution (Sigma-Aldrich, Darmstadt, Germany) was applied to the culture surface for coating, which was previously sterilized under a UV lamp along with the whole device. The collagen solution was left overnight to bind to the surface and then aspirated to allow the device to dry. After coating, the device was washed with 1× PBS before cell loading. Huh7 cells were seeded into both circular and elliptical microfluidic devices at a density of 675 cells·mm^−2^. The density was based on previous trials of optimal cell density for the devices and manufacturer instructions from a similar organ-on-a-chip device (MesoBioTech, Paris, France). Detailed calculations are given in the Supplementary Materials section. The cells were cultured for 24 h in the devices and in control plates. 

#### 2.4.3. Microfluidic Flow Experiments

The microfluidic experimental setup was composed of a chip and a microfluidic syringe pump (Aladin, World Precision Instruments, Tregoland OU, Tallinn, Estonia) for the accurate control and adjustment of fluid flow rates of media. Dulbecco’s modified Eagle’s medium (DMEM) filled in the syringe was supplied to the chips through the inlets of the microfluidic devices. The effluent, which may contain metabolites and secreted proteins, was subsequently collected from the chip outlet.

Experiments were conducted for both devices, i.e., the two chips with circular and elliptical chambers. The first experiment pertains to filling-time measurements at a flow rate of 15 μL·min^−1^, using DI water. Filling time was measured with a stopwatch, and the experiment was repeated five times for each chip to obtain average values and compare them with simulation results.

In order to assess cell viability and functionality, the second fluidic experiment was conducted on cell seeded microfluidic devices for 24 h in continuous flow mode. The two microfluidic devices and the control plates were monitored for cell growth over the 24 h period. After 24 h, the chips containing the cells were connected to a perfusion system, ensuring a secure and leak-proof connection. The medium flow rate was set to 3 μL·min^−1^.

### 2.5. Biocompatibility and Cell Growth Assessment in Circular and Elliptical Microfluidic Devices

#### 2.5.1. Sample Collection

Samples of the perfusion medium were collected at regular intervals to monitor changes in cellular functionality over a 24 h period. The perfused medium was collected in 300 μL volume fractions every 180 min. After completion of the flow experiments, a total of five samples representing different time points over a 24 h period were selected for further cell functional analysis.

#### 2.5.2. Cell Functionality Assessment

To assess the metabolic activity of cells, ELISA assays were used to determine the secretion rates of hepatic biomarkers in microfluidic devices under perfusion. Samples obtained over 24 h of continuous flow were analyzed for albumin and urea secretion rates via ELISA according to manufacturers’ instructions (Thermo Fisher Scientific, Waltham, MA, USA). All samples were analyzed in duplicates. For urea detection, the absorbance values of the DMEM control were subtracted from the data points to compensate for the background readings of the components of the DMEM medium. Measurements for both assays were performed at 450 nm using a Varioskan Flash microplate reader (Thermo Scientific, Waltham, MA, USA). 

#### 2.5.3. Cell Viability and Microscopy Imaging

Cell viability was assessed using a Live/Dead Cell Viability Assay Kit (Sigma-Aldrich, Darmstadt, Germany) following the manufacturer’s instructions. In summary, cells were stained with calcein AM and propidium iodide to label live and dead cells, respectively. Cell viability was assessed both before and at the end of the flow experiment. Optical and fluorescent images of the cell culture were obtained using a ZOE Fluorescent Cell Imager (Bio-Rad, Hercules, CA, USA).

## 3. Results and Discussion

We begin our discussion in this section with the presentation of CFD simulation results, including mesh convergence studies. We then proceed with experimental results on filling time measurement and their comparison to computational results. Hepatocytes growth and cell viability assessments are also discussed herein.

### 3.1. CFD Simulation Results

To investigate the filling time and filling flow characteristics of the circular and elliptical microfluidic devices, simulations were carried out using COMSOL Multiphysics and ANSYS Fluent software packages. The use of two different computational tools allow for additional comparison between the two approaches to verify their alignment or divergence. Additionally, the accuracy of the two modeling approaches with the conducted experiments can be benchmarked. Thus, this two-fold comparison strengthens the validity of the modeling approaches and the presented results, while at the same time identifying the range of expected deviations between simulated and experimental results. According to the results, although some slight differences exist between the two simulations, they are in general agreement with the elliptical design, clearly outperforming the circular one.

**Figure 4:**
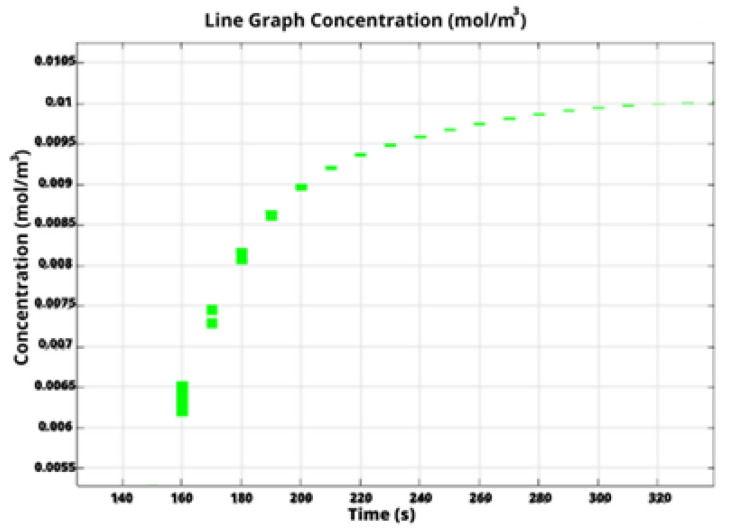
Water concentration at the outlet boundary versus time for the design with a circular chamber.

Specifically, in COMSOL Multiphysics, we calculate the filling time through monitoring the concentration of water at the outlet boundary. Figure 4 depicts water concentration at the outlet versus time for the liver-on-a-chip with a circular chamber. The initial water concentration at the outlet is obviously zero, subsequently increasing until it reaches a steady state with a constant value equal to the inlet water concentration. This indicates that the chip was completely filled and the flow has been stabilized.

**Figure 5:**
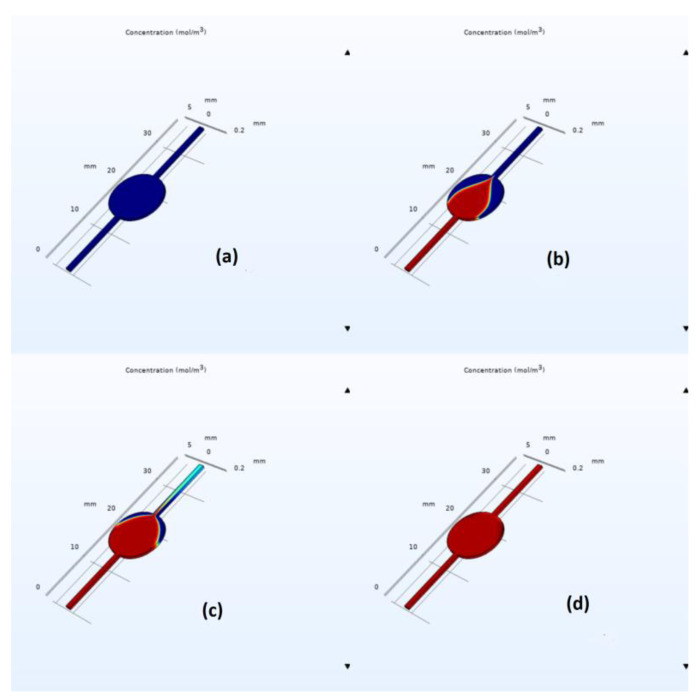
Visual representation of the filling process for the circular chamber at an inlet flow rate of 15 µL/minute at (**a**) 0, (**b**) 100, (**c**) 220, and (**d**) 320 s. The blue color corresponds to air, and the red color corresponds to water flowing from the inlet.

**Figure 6:**
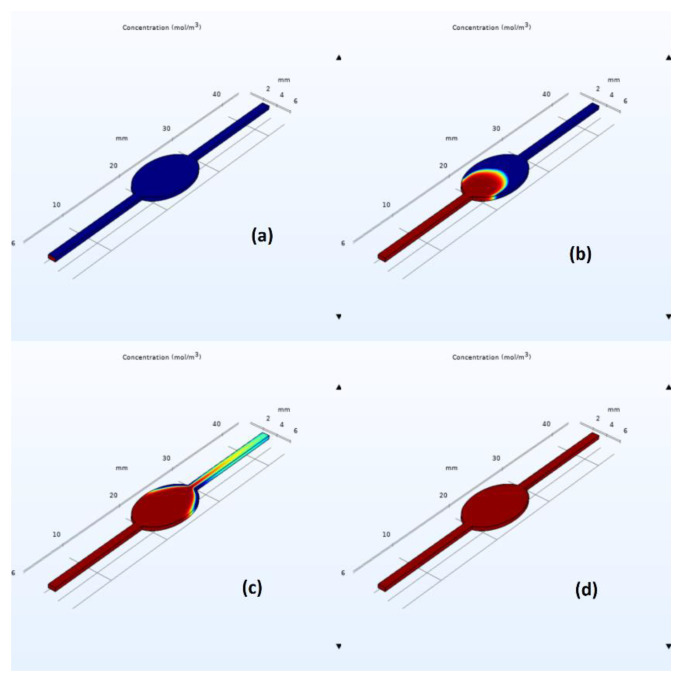
Visual representation of the filling process for the elliptical chamber with an inlet flow rate of 15 µL/minute at (**a**) 0, (**b**) 70, (**c**) 150, and (**d**) 220 s. The blue color corresponds to air, and the red color corresponds to water flowing from the inlet.

COMSOL simulation snapshots for the circular and elliptical chambers are depicted in Figure 5 and Figure 6, respectively. As we can easily observe from these figures, the elliptical design exhibits a faster filling time, with the estimated filling time values being equal to 320 s for the circulate chamber and 220 s for the elliptical one; see the corresponding mesh convergence results in Table 1. Furthermore, flow velocity in the middle part of the elliptical chip was found to be higher than that of the circular one with an average value of 1.4 × 10^−4^ and 0.85 × 10^−4^ m/s, respectively. This is due to the well-documented effect of flow disturbance when a sudden expansion is met, followed by a sudden constriction at the end of the circular chamber. However, in the elliptical case, the expansion and constriction are less abrupt, leading to better flow characteristics and reduced air bubble entrapment. Simulations indicated air entrapment in both cases; however, the amount of trapped air was significantly lower in the elliptical design.

**Figure 7:**
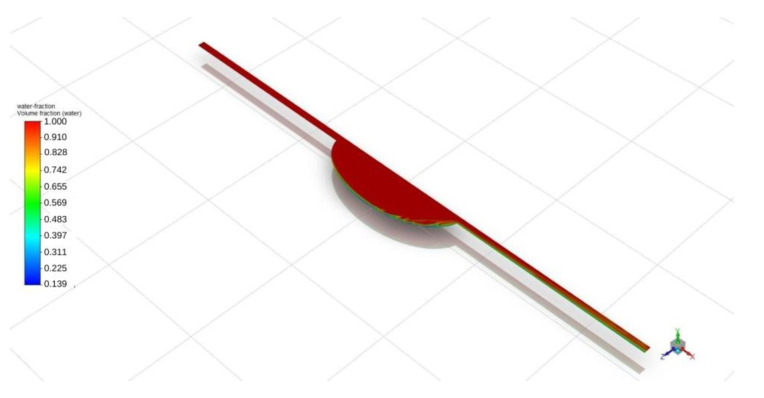
Elliptical chip air entrapment.

**Figure 8:**
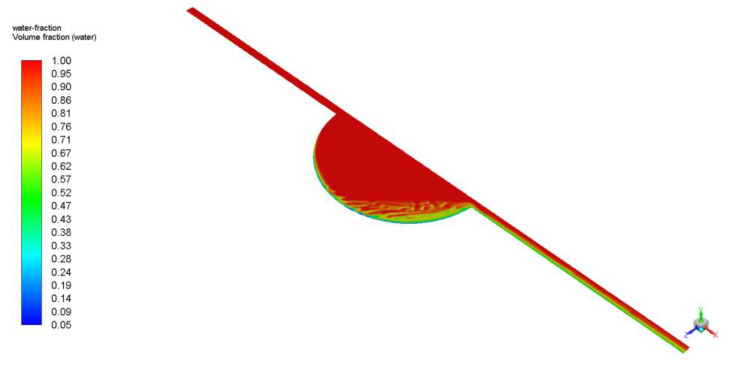
Circular chip air entrapment.

Similar qualitative and quantitative results were acquired when using ANSYS Fluent; see Figure 7 and Figure 8. Specifically, for the circular design, filling time was estimated at 350 s, with noticeable air entrapment at the lateral walls of the central chamber. Once again, simulations revealed that air entrapment occurred throughout the filling process for both circular and elliptical liver-on-a-chip designs. However, the quantity of air entrapment in the elliptical form was found to be substantially smaller than in the circular design. The circular design trapped much of the air at the corners of the circular chamber. On the contrary, the air was able to exit, to a large extent, from the elliptical chamber due to the more streamlined transition between the channels and the central chamber. As a consequence, the elliptical shape demonstrated a more uniform filling pattern with less air entrapment on the channel walls.

As filling time is clearly reduced and, more importantly, since decreased air entrapment is observed, the elliptical design is portrayed as a better design for the liver-on-a-chip platform. Mesh convergence results are presented in the Table 2. However, experimental validation is required to demonstrate the final efficiency of the elliptical design and examine its applicability for various liver-on-a-chip applications.

### 3.2. Filling Time and Shear Stress Analysis of Microfluidic Devices

Filling time measurement provides us with valuable information about flow dynamics within the liver-on-a-chip system and helps in identifying potential problems and optimizing experimental conditions and designs. Five experimental trials were carried out for both circular and elliptical chambers, and their average values were compared to the computational simulation results from ANSYS and COMSOL (Table 3). The actual flow rate was measured with a flow meter and found to be consistent with the desired flow rate. The flow rate was constant throughout the experiment, indicating that the device provided stable flow conditions.

Additionally, to calculate shear stress values on the walls of both microfluidic devices, five points along the culture chamber were selected and compared (Table 4). As expected, the highest values of shear stress in both chambers are observed at the inlet and outlet locations, with values decreasing gradually towards the plane perpendicular to the flow (90 degrees). Even though there is an order of magnitude difference between wall shear stress values for the elliptic and circular designs, the values remain low for both cases and should not affect the cell culture morphology. In general, physiologically relevant shear stress values inside microfluidic devices lie in the 0 to 0.03 dyn/cm^2^ (3 mPa) range based on a study conducted with intestinal epithelial cells [21]. Tiles et al. have reported that shear stress rates above (5 dyn/cm^2^) 0.5 Pa reduced hepatocyte function [22]. In this work, the highest shear stress generated in the chamber remains much lower than the highest shear stress values reported, suggesting that there is no detrimental mechanical effect on cell morphology in both devices.

### 3.3. Evaluation of Cell Viability in Static and Continuous Modes

The suitability of microfluidic devices composed of gas-permeable and non-permeable materials was investigated. In our initial experiments, we constructed the circular and elliptical chip designs entirely out of cyclic-olefin copolymer (COC), but a hybrid polydimethylsiloxane (PDMS) and COC design was chosen, where the gas-permeable PDMS material served as the top layer of the chips. The biocompatibility and adsorption characteristics of PC, COC, and PDMS have been previously reported in the literature [23,24].

To assess the biocompatibility of the device material, hepatocyte growth was studied in microfluidic devices made solely of COC and the COC–PDMS mixture. Huh7 cells were cultured for 24 h in static conditions, and cell growth in the devices was assessed via optical microscopy and cell viability analysis. In the devices made entirely out of COC, no cell attachment to the surface was observed (see Figure S3). As expected, cell culture in the devices made out of PDMS and COC resulted in successful cell growth and attachment to chip surfaces.

**Figure 9:**
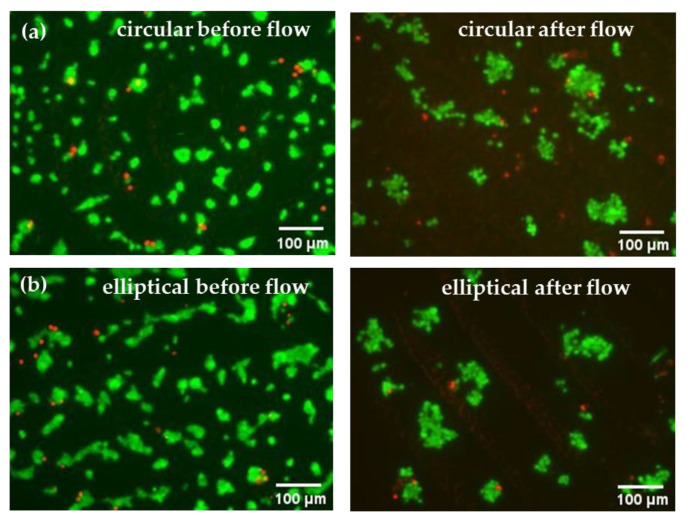
Live–dead cell imaging results of the Huh7 hepatoma cell line cultured in microfluidic devices in static and after 24 h of continuous flow: (**a**) circular and (**b**) elliptical microfluidic device in static mode (**left**) and after 24 h of continuous flow (**right**). Green (Calcein-AM)—live cells; red (Propidium Iodide)—dead cells.

After 24 h of culturing Huh7 cells in the microfluidic devices under static conditions, live/dead assay analyses were carried out, which confirmed successful cell attachment in both circular and elliptical COC–PDMS microfluidic devices. Resulting images can be seen from the left side of Figure 9. Compared to the control batch (Figure S4), the cell density in the microfluidic devices was smaller; however, this is expected as most non-adherent or dead cells on the device surfaces can be washed away with the effluent. Additionally, some loosely attached cells can be removed under higher shear stress and applied pressure in the microchannels in comparison to the case of static well plates with much higher volumes. Overall, minimal presence of dead cells was observed in the static microfluidic culture, which confirmed that the devices were biocompatible and more suitable for further use in this study. According to the literature, PDMS is known for its excellent gas permeability and biocompatibility, which ensures gas exchange and also promotes cell attachment [24]. The confirmation of cell attachment through live/dead assay microscopy images for both circular and elliptical devices also indicated that the shape or geometry of the devices did not significantly affect cell attachment. As can be seen from the left images shown in Figure 9, cell cultures in both devices show comparable numbers and growth of cells.

Perfusion studies were carried out for both the circular and elliptical device simultaneously through seeding Huh7 cells and passing the medium through the device at a controlled constant flow rate for 24 h. The perfusion medium was pumped at a flow rate of 3 μL·min^−1^ to assess the ability of the devices to culture cells over time. Although the flow rate was consistent throughout the experiment, an elliptical microfluidic chip offered easier handling and no air bubble entrapment at the first loading stage of the media into the devices. These observations were consistent with CFD simulation results, which indicated decreased air bubble entrapment in the elliptical device as opposed to its counterpart. Having established a stable flow, both microfluidic devices caused no leakage or visible air bubbles, which indicated that the experimental setup was properly sealed and functioned as expected. This is important as it ensures that the cells receive a continuous supply of fresh media and nutrients. The supernatants were collected every 180 min throughout the flow experiment for further biomarker studies.

Similarly to static conditions, cells after the 24 h perfusion studies were evaluated using live/dead assay microscopy analysis of their viability. Figure 9 (right) shows live/dead staining images of cells in the channels of the device after 24 h of continuous flow, where live (green) cells predominate over dead cells, suggesting that cell viability was stable, and perfusion did not inhibit cell growth in both devices. Nevertheless, results indicated that perfusion affected Huh7 cell morphology, leading to cell attachment to each other and cluster formation. Although the number of attached cells are reduced after the 24 h flow, overall, based solely on microscopy images, both circular and elliptical devices were found to be suitable for perfusion studies and hepatocyte culture. 

### 3.4. Biomarker Level Studies

**Figure 10:**
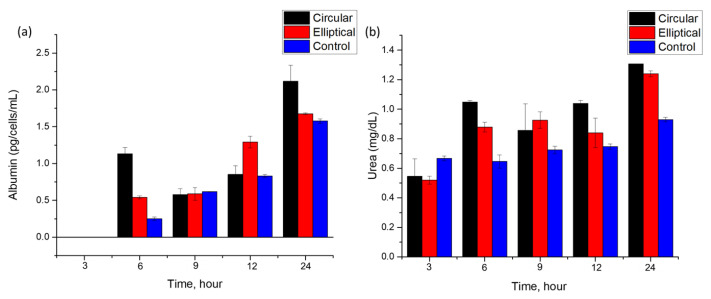
(**a**) Albumin and (**b**) urea secretion levels produced by Huh7 cultured in the circular and elliptical microfluidic devices under continuous flow mode and in control samples cultured in static conditions.

Although microscopy images confirmed the presence of attached and growing Huh7 cells, the health of cells in the microfluidic devices needed to be evaluated over time. To assess and compare the suitability of the circular and elliptical microfluidic devices for culturing healthy cell culture under perfusion, a constant flow rate of 3 μL·min^−1^ was maintained and samples of eluents were collected every 3 h up to 24 h. The functionality of the liver cells within the microfluidic device was evaluated through measuring the levels of key biomarkers like albumin and urea. These biomarkers indicate the metabolic activity and functionality of the hepatoma cell lines [25,26]. The secretion levels of albumin and urea were evaluated at various time intervals. For both devices and control samples, a total of five samples were obtained with a time interval of 3 h, and the last sample was taken after a total culture time of 24 h under perfusion (Figure 10).

Both circular and elliptical microfluidic devices cultured with Huh7 cells showed relatively stable albumin secretion up to 24 h, as shown in Figure 10a. The first 3 h sample showed a negligible amount of albumin levels. The presence of albumin was first observed in a 6 h sample for both devices and control. Particularly, in the circular device, a sharp increase has been observed for the 6 h time mark. This increase and fluctuation in the albumin secretion rate over the experiment duration may indicate fluctuations in metabolic activity and cell functionality over time, which are in agreement with previous work [13,27,28]. The absence of albumin in the first sample and observed albumin levels later may reflect the adaptation of the cells to the perfusion culture and flow conditions [13,27].

Albumin concentrations were calculated in units of pg·cell^−1^·mL^−1^·h^−1^, and the doubling time of cells was taken into account for analysis. The Huh7 hepatoma cell line typically has a doubling time of 24 h as reported [29], which accounted for the final cell number used for the post-24 h culture of cells in static conditions. Albumin levels secreted by cells in the circular and elliptical devices under continuous flow modes were determined to be (0.26 ± 0.02) pg·cell^−1^·mL^−1^·h^−1^ and (0.24 ± 0.01) pg·cell^−1^·mL^−1^·h^−1^, respectively. For control samples cultured in static conditions, the level of albumin secreted by the cells was about (0.06 ± 0.001) pg·cell^−1^·mL^−1^·h^−1^, which was much lower than the levels of the biomarkers obtained from the microfluidic devices. The difference may be due to the enhancement of the albumin secretion rate by continuous flow in comparison to static culture. Additionally, considering that based on microscopy images, some cells may have been detached due to shear stress in the microchannels, the determined albumin levels potentially could be even higher than the ones reported in the study. The results are in line with previously reported values of albumin secretion rates, where the average value of albumin secretion rate was (0.11 ± 0.02) pg·cell^−1^·mL^−1^·h^−1^ [13]. Different batch and cell sources of the Huh7 cell line can be a cause of slight variation. Overall, on average, both devices consistently showed higher albumin levels over time than the values of albumin in control samples. In addition to the easier handling and absence of air entrapment offered by the elliptical microfluidic chip, this study showed at least as equal albumin secretion rates of cells cultured in the elliptical device as in the cells cultured in its counterpart circular chip.

In addition to the albumin secretion rate, another biomarker, urea, has been chosen for the analysis of cell culture quality and growth in the microfluidic devices. Figure 10b shows the dependence of urea concentration found in the collected samples at various time points up to 24 h. Results show an increase in the secretion rate of urea by cells cultured in both devices. Cells consistently demonstrated urea secretion over the experimental time. The minimum values of urea in the collected samples were similar for both devices ~ (0.5 ± 0.02) mg·dL^−1^. These values are comparable to those reported in the previously published literature (0.43 ± 0.04) mg·dL^−1^ [13]. An increase over time was also reported previously. Over 24 h of perfusion, urea levels doubled in both devices, suggesting that the perfusion culture in the microfluidic devices was functioning efficiently. The values are comparable to the urea secretion levels observed in the literature [30], which reported a time-dependent increase in urea secretion over time when Huh7 cells were cultured in 2D monolayers.

## 4. Conclusions

The microfluidic platforms for hepatocyte culture investigated in this work can effectively mimic certain organ characteristics, which allows researchers to accelerate drug development and reduce reliance on animal models. The study focused on the design and modeling of COC–PDMS microfluidic devices and evaluated their biocompatibility, filling behavior, and cell functionality under perfusion. Multiple CFD simulations on Comsol Multiphysics (5.5) and Ansys (2022r2) software and experiments were conducted to analyze the filling behavior of the previously reported circular design and the newly proposed elliptical microfluidic platform. The elliptical design, with its slightly lower volume and streamlined geometry, demonstrated reduced air entrapment compared to the circular design, emphasizing the significance of device geometry in facilitating efficient fluid flow within the microchannels. The biocompatibility of the microfluidic devices was assessed through seeding Huh7 cells into the chips. The results showed that both the circular and elliptical microfluidic devices provide a suitable microenvironment for cell growth and function. The cells exhibited typical hepatocyte cell morphology, and the device showed minimal cell death after 24 h, highlighting its capability to support the viability and functionality of liver cells under controlled flow conditions. The simulation results obtained from Comsol and Ansys have proven to be a valuable tool to accurately model flow dynamics in a liver-on-a-chip microfluidic device. Also, these results have not only validated the device’s design but also offered valuable guidance for optimizing experimental conditions in liver-on-a-chip studies. 

Overall, the analysis of fluid flow behavior, filling characteristics, and cell functionality in liver-on-a-chip devices contributes to the advancement of design optimization and performance enhancement. The study’s findings provide valuable insights into the potential applications of liver-on-a-chip platforms, offering an alternative approach to animal testing in drug development.

## Figures and Tables

**Table 1:** Mesh convergence results for filling time in COMSOL.

Number of Elements	Filling Time, s	Deviation, %
Circular design		
23,509	345	-
36,995	329	4.6
116,029	320	2.8
Elliptical design		
23,313	234	-
44,934	211	9.8
120,528	220	4.3

**Table 2:** Mesh convergence results for air volume fraction in Ansys.

Elements	Air Volume Fraction	Deviation, %
4815	0.006557923	N/A
30,260	0.01439314	119.5
76,118	0.01806330	25.5
136,792	0.01722741	4.6
239,460	0.01670033	3.1

**Table 3:** Experimental and computational data (Comsol, Ansys) comparison.

Trial	Filling Time Circular Design, Seconds	Filling Time Elliptical Design, Seconds
1	315	203
2	264 (water trapped, big bubble formation)	170
3	307	198
4	299	209
5	290	215
Experimental average	295	199
COMSOL	320	220
ANSYS	300–320	210–230
Deviation, %	8.47%	10.55%

**Table 4:** Wall shear stress values along the chamber at symmetrical points (Pa).

Symmetrical Points	Elliptical	Circular
Inlet	1.04 × 10^−4^	6.97 × 10^−5^
45 degrees	1.28 × 10^−5^	4.72 × 10^−6^
90 degrees	1.17 × 10^−5^	4.08 × 10^−6^
135 degrees	1.27 × 10^−5^	4.52 × 10^−6^
Outlet	1.03 × 10^−4^	7.16 × 10^−5^

## Data Availability

Data are contained within the article or Supplementary Materials.

## Supplementary Materials

The following supporting information can be downloaded at: https://www.mdpi.com/article/10.3390/bios13070754/s1, Figure S1: Circular liver-on-a-chip drawings; Figure S2: Elliptical liver-on-a-chip drawings; Figure S3: Optical images of the Huh7 hepatoma cell line cultured in microfluidic devices made with (a) COC and (b) COC-PDMS; Figure S4: Huh7 cell control samples; Figure S5: Biomarker level studies: Albumin standard curve; Figure S6: Biomarker level studies: Urea standard curve; Surface area calculations: circular microfluidic device, elliptical microfluidic device; Table S1: Albumin absorbance values of both microfluidic devices and control samples; Table S2: Albumin absorbance values of both microfluidic devices and control samples subtracted from the blank (media); Table S3: Albumin concentration values in pg/ml/cell for both microfluidic devices and control samples; Table S4: Urea absorbance values of both microfluidic devices and control samples; Table S5: Urea Absorbance values of both microfluidic devices and control samples subtracted from the blank (media); Table S6:Urea concentration values in mg/dL for both microfluidic devices and control samples.
