# Supplementary material for: Design, Simulation, and Evaluation of Polymer-Based Microfluidic Devices via Computational Fluid Dynamics and Cell Culture “On-Chip”
Source: Biosensors (Basel). 2023 Jul 22;13(7):754. doi: 10.3390/bios13070754 (PMC10377015; doi:10.3390/bios13070754)
Supplement: Supplementary file 1 [file biosensors-13-00754-s001.zip › biosensors-2470521-supplementary.pdf]

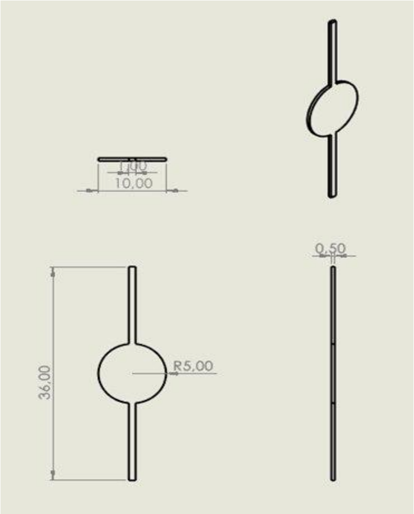

Figure S1. Circular liver-on-a-chip drawings

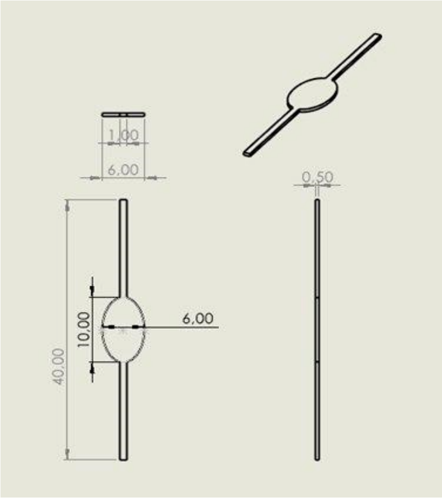

Figure S2. Elliptical liver-on-a-chip drawings

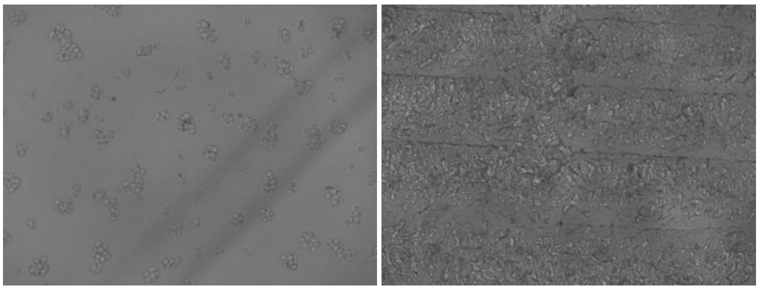

Figure S3. Optical images of the Huh7 hepatoma cell line cultured in microfluidic devices made with (a) COC and (b) COC-PDMS

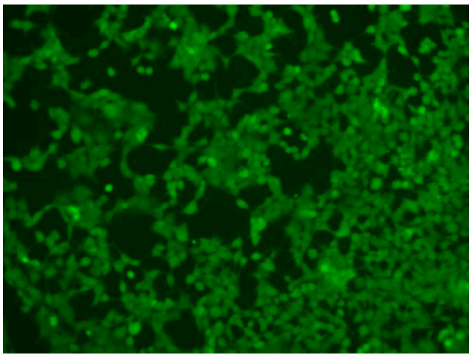

Figure S4. Control samples. Live-dead staining images of the Huh7 cells cultured in standard collagen-coated 24 well plates

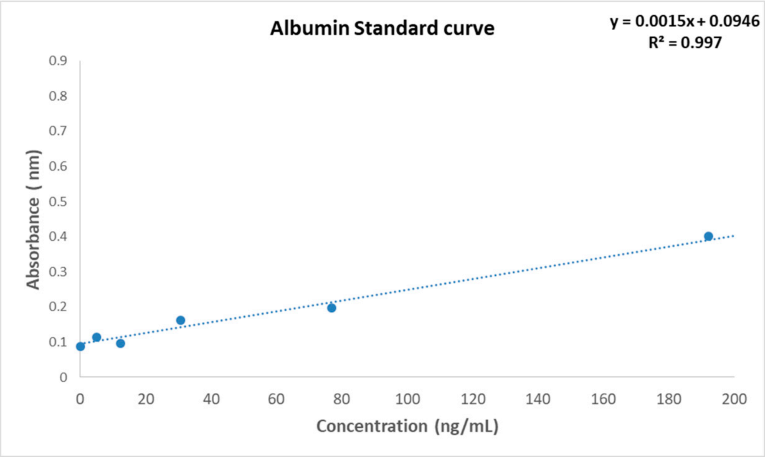

Figure S5. Biomarker level studies: Albumin standard curve

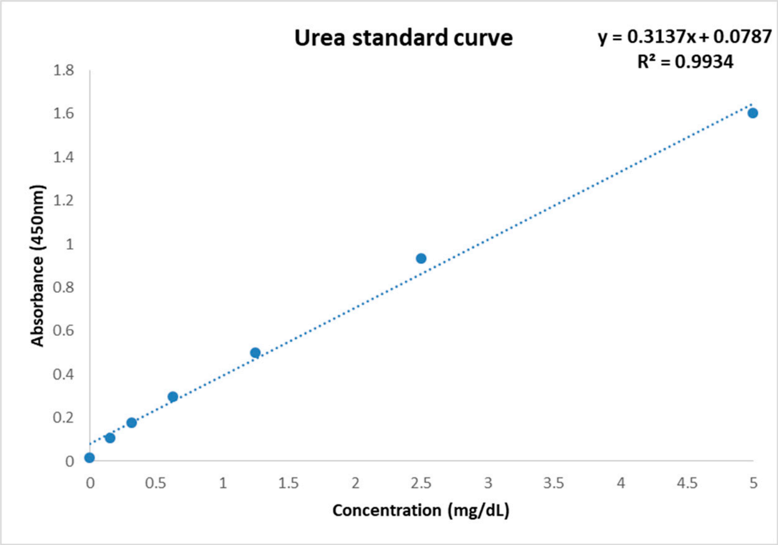

Figure S6. Biomarker level studies: Urea standard curve

<u>Surface area calculations</u>

A microfluidic device with a circular chamber

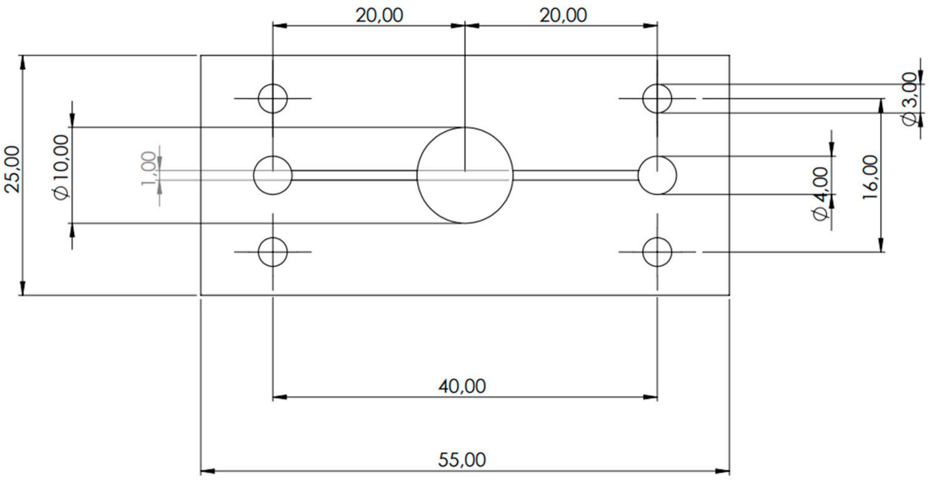

Inlet-chamber-outlet:

$$A_{chip} = A_{inlet} + A_{circular\ chamber} + A_{outlet}$$
$$A_{chip} = \pi * 2^2 + \pi * 5^2 + \pi * 2^2 = 33\pi = 103.67\ mm^2$$

Inlet-chamber-outlet-channel:

$$A_{chip} = A_{inlet} + A_{circular\ chamber} + A_{outlet} + A_{channel} - A_{segments}$$
$$A_{segments} = A_{inlet\ segment} + A_{chamber\ segment} + A_{outlet\ segment}$$
$$A_{inlet\ segment} = 1.94 + 0.04 = 1.98\ mm^2$$
$$A_{chamber\ segment} = 2(4.97 + 0.018) = 9.976\ mm^2$$
$$A_{outlet\ segment} = 1.94 + 0.04 = 1.98\ mm^2$$
$$A_{channel} = 40\ mm^2$$
$$A_{inlet-chamber-outlet} = 33\pi$$
$$A_{chip} = 33\pi + 40 - (1.98 + 9.976 + 1.98) = 129.74\ mm^2$$

Seeding density = $A_{inlet-chamber-outlet}$x 675 cells·mm-2 = 33π x 675 cells·mm-2 = 69943,5 ≃ 70000 cells

A microfluidic device with an elliptical chamber

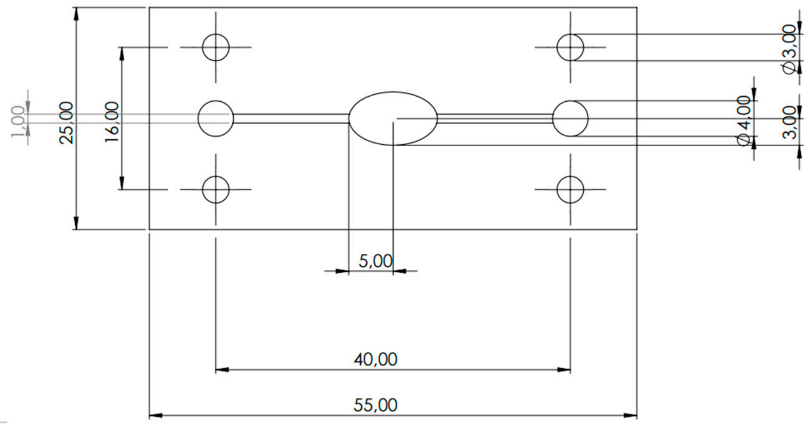

Inlet-chamber-outlet:

$$A_{chip} = A_{inlet} + A_{elliptical\ chamber} + A_{outlet}$$
$$A_{chip} = \pi * 2^2 + 47.12 + \pi * 2^2 = 47.12 + 8\pi = 72.25\ mm^2$$

Inlet-chamber-outlet-channel:

$$A_{chip} = A_{inlet} + A_{elliptical\ chamber} + A_{outlet} + A_{channel} - A_{segments}$$
$$A_{segments} = A_{inlet\ segment} + A_{chamber\ segment} + A_{outlet\ segment}$$
$$A_{inlet\ segment} = 1.94 + 0.04 = 1.98\ mm^2$$
$$A_{chamber\ segment} = 2(4.93 + 0.047) = 9.954\ mm^2$$
$$A_{outlet\ segment} = 1.94 + 0.04 = 1.98\ mm^2$$
$$A_{channel} = 40\ mm^2$$
$$A_{inlet-chamber-outlet} = 47.12 + 8\pi$$
$$A_{chip} = 47.12 + 8\pi + 40 - (1.98 + 9.954 + 1.98) = 98.34\ mm^2$$

Seeding density = $A_{inlet-chamber-outlet}$x 675 cells·mm-2 = 72,24 x 675 cells·mm-2 = 48762 ≃ 48800 cells

Table S1. Albumin absorbance values of both microfluidic devices and control samples

**Circular 1**	**Circular 2**	**Mean**	**SD**	**Control 1**	**Control 2**	**Mean**	**SD**	**Elliptical 1**	**Elliptical 2**	**Mean**	**SD**
0.1394	0.1345	0.1369	0.0034648	0.094465	0.099982	0.097224	0.0039011	0.121565	0.1256	0.1235825	0.0028531
0.393154	0.42054	0.4068	0.0193648	0.247654	0.263178	0.255416	0.0109771	0.23959	0.234854	0.2372222	0.0033485
0.270054	0.29554	0.2827	0.0180213	0.405543	0.407969	0.406756	0.0017154	0.253947	0.2353	0.2446237	0.0131857
0.362588	0.324856	0.3437	0.0266808	0.488297	0.500424	0.494360	0.0085750	0.363305	0.34574	0.3545228	0.0124207
0.6617	0.5924	0.6270	0.0490025	0.809012	0.791512	0.800262	0.0123743	0.413344	0.415742	0.414543	0.0016956

Table S2. Albumin absorbance values of both microfluidic devices and control samples subtracted from the blank (media)

**Circular 1**	**Circular 2**	**Control 1**	**Control 2**	**Elliptical 1**	**Elliptical 2**	**Media**
0.0789722	0.0740722	0.0340381	0.0395551	0.0611372	0.0651722	0.0604278
0.3327262	0.3601122	0.1872262	0.2027502	0.1791622	0.1744266	
0.2096262	0.2351122	0.3451152	0.3475412	0.1935197	0.1748722	
0.3021606	0.2644282	0.4278692	0.4399962	0.3028778	0.2853122	
0.6012722	0.5319722	0.7485842	0.7310842	0.3529162	0.3553142	

Table S3. Albumin concentration values in pg/ml/cell for both microfluidic devices and control samples

**Circular 1**	**Circular 2**	**Mean**	**SD**	**Control 1**	**Control 2**	**Mean**	**SD**	**Elliptical 1**	**Elliptical 2**	**Mean**	**SD**
-0.02027	-0.02757	-0.023	0.0051559	-0.04756	-0.04308	-0.04532	0.003168	-0.06723	-0.05860	-0.062919	0.006096
0.35733	0.398083	0.3777	0.028816	0.038428	0.044732	0.041580	0.0044578	0.18496	0.17484	0.1799081	0.0071552
0.17414	0.212071	0.1931	0.026817	0.068365	0.069022	0.068694	0.0004644	0.215647	0.175800	0.1957238	0.0281755
0.31184	0.25569	0.2837	0.039703	0.068077	0.070540	0.069308	0.0017412	0.449325	0.411790	0.4305581	0.026540
0.22708	0.196148	0.2116	0.021876	0.066600	0.064823	0.065711	0.001256	0.166874	0.16841	0.1676431	0.0010869

Table S4. Urea absorbance values of both microfluidic devices and control samples

**Circular 1**	**Circular 2**	**Mean**	**SD**	**Control 1**	**Control 2**	**Mean**	**SD**	**Elliptical 1**	**Elliptical 2**	**Mean**	**SD**

1.18019	1.23271	1.206	0.0371372	1.24123	1.24833	1.24478	0.0050204	1.19266	1.20432	1.19849	0.0082448
1.36655	1.36221	1.3643	0.0030688	1.24781	1.22822	1.238015	0.0138522	1.31848	1.3042	1.31134	0.0100974
1.34386	1.26462	1.3042	0.0560311	1.25673	1.26822	1.262475	0.0081246	1.3382	1.31375	1.325975	0.0172887
1.36597	1.35638	1.3611	0.0067811	1.26562	1.2735	1.26956	0.0055720	1.32083	1.27691	1.29887	0.0310561
1.44499	1.44569	1.4453	0.0004949	1.3236	1.33022	1.32691	0.0046810	1.42026	1.42893	1.424595	0.0061306

Table S5. Urea absorbance values of both microfluidic devices and control samples subtracted from the blank (media)

**Circular 1**	**Circular 2**	**Control 1**	**Control 2**	**Elliptical 1**	**Elliptical 2**	**Media**
0.22346	0.27598	0.2845	0.2916	0.23593	0.24759	0.95673
0.40982	0.40548	0.29108	0.27149	0.36175	0.34747	
0.38713	0.30789	0.3	0.31149	0.38147	0.35702	
0.40924	0.39965	0.30889	0.31677	0.3641	0.32018	
0.48826	0.48896	0.36687	0.37349	0.46353	0.4722	

Table S6. Urea concentration values in mg/dL for both microfluidic devices and control samples

**Circular 1**	**Circular 2**	**Mean**	**SD**	**Control 1**	**Control 2**	**Mean**	**SD**	**Elliptical 1**	**Elliptical 2**	**Mean**	**SD**
0.46145	0.628881	0.5451	0.118384	0.65604	0.678673	0.667357	0.0160040	0.50121	0.538380	0.5197959	0.0262826
1.055530	1.041695	1.0486	0.0097827	0.67701	0.614568	0.645792	0.0441575	0.902295	0.856773	0.8795345	0.0321883
0.983200	0.730602	0.8569	0.1786137	0.705451	0.742078	0.723764	0.0258994	0.965157	0.887217	0.9261874	0.0551124
1.053681	1.023111	1.0383	0.0216166	0.733790	0.758909	0.746350	0.0177621	0.909786	0.769780	0.8397832	0.0989994
1.30557	1.307810	1.3066	0.0015778	0.918616	0.939719	0.929167	0.0149220	1.226745	1.254383	1.2405642	0.0195429
